# Applications of Thiol-Ene Chemistry for Peptide Science

**DOI:** 10.3389/fchem.2020.583272

**Published:** 2020-11-12

**Authors:** Mark D. Nolan, Eoin M. Scanlan

**Affiliations:** School of Chemistry, Trinity College Dublin, Trinity Biomedical Sciences Institute, Dublin, Ireland

**Keywords:** peptides, bioconjugation, radical, thiol (-SH), protein

## Abstract

Radical thiol-ene chemistry has been demonstrated for a range of applications in peptide science, including macrocyclization, glycosylation and lipidation amongst a myriad of others. The thiol-ene reaction offers a number of advantages in this area, primarily those characteristic of “click” reactions. This provides a chemical approach to peptide modification that is compatible with aqueous conditions with high orthogonality and functional group tolerance. Additionally, the use of a chemical approach for peptide modification affords homogeneous peptides, compared to heterogeneous mixtures often obtained through biological methods. In addition to peptide modification, thiol-ene chemistry has been applied in novel approaches to biological studies through synthesis of mimetics and use in development of probes. This review will cover the range of applications of the radical-mediated thiol-ene reaction in peptide and protein science.

## Introduction

**Figure 1:**
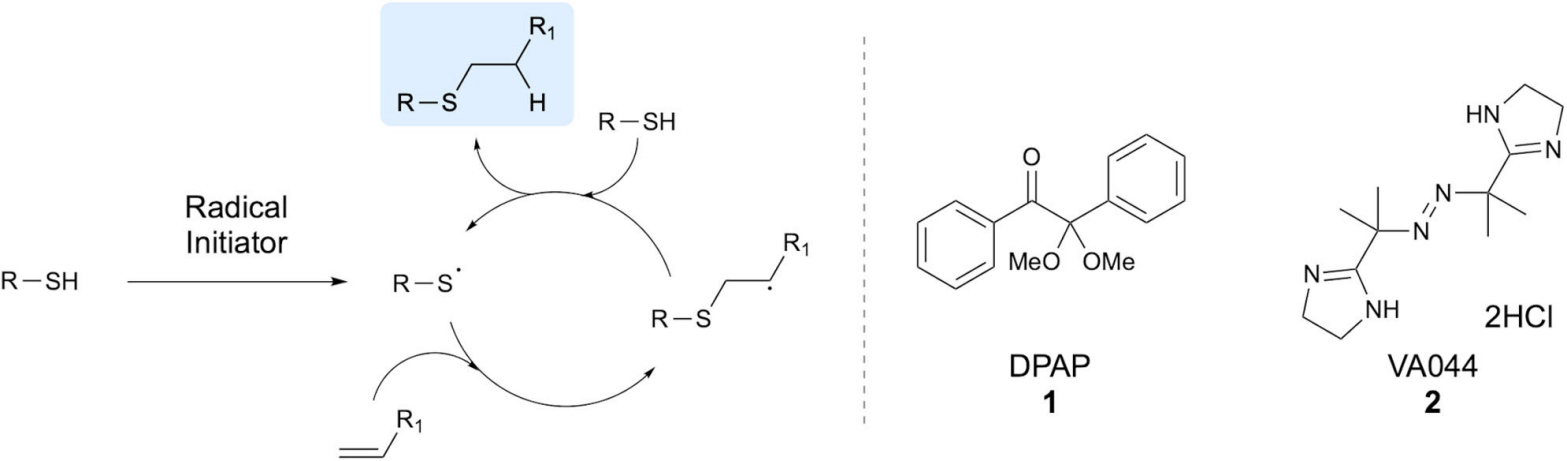
Radical cycle of thiol-ene coupling and structures of two common initiators. The radical cycle for a generalized thiol-ene coupling reaction is shown, along with the commonly applied radical initiators DPAP and VA044.

Since its discovery by Posner (1905), the thiol-ene reaction has found many diverse applications in synthetic chemistry. This efficient process involves the generation of a thiyl radical from a thiol, which subsequently undergoes anti-Markovnikov addition to an alkene, furnishing a carbon-centered radical. The carbon-centered radical abstracts a hydrogen from another molecule of thiol to give the thioether product, along with facilitating propagation of the radical cycle through generation of a new thiyl radical (Figure 1). Thiyl radicals are readily formed through homolytic cleavage of the sulfhydryl S-H bond due to low bond dissociation energies of around 87 kcal mol^−1^ (Dénès et al., 2014). However, both thermal and photochemical radical initiators are often applied to aid in radical formation. In particular, applications of the thiol-ene reaction in peptide chemistry often make use of 2,2-dimethoxy-2-phenylacetophenone (DPAP) **1** or the water-soluble 2,2'-azobis[2-(2-imidazolin-2-yl)propane]-dihydrochloride (VA044) **2** radical initiator (Figure 1). Radical thiol-ene chemistry has been demonstrated for many applications including, but not limited to thiosugar synthesis and carbohydrate chemistry (McSweeney et al., 2016), polymerisations (Hoyle et al., 2004), surface chemistry (Hoyle and Bowman, 2010), synthetic chemistry (Dénès et al., 2014) and in peptide chemistry, the latter being the focus of this review.

The thiol-ene reaction, or thiol-ene coupling (TEC) is considered a “click” reaction (Hoyle and Bowman, 2010) as originally defined by Shapless in Kolb et al. (2001). Notable features of such “click” reactions include high yields, lack of side products, compatibility with aqueous conditions and orthogonality to many other synthetic reactions. These “click” characteristics prove ideal for application to peptide science. Whilst high yields mean valuable starting material is not wasted and the lack of side products allows easier purification, the compatibility with aqueous conditions is ideal for peptide dissolution and reaction orthogonality allows for minimal or no use of side-chain protecting groups. In addition, the thioether linkage produced in the reaction is stable in a range of chemical environments, tolerating pH ranges.

The absence of any requirement for metal catalysts in the thiol-ene reaction is of particular advantage in peptide chemistry. This avoids the need for removal of often toxic metals for biological applications. This is in contrast to the widely utilized copper-catalyzed azide-alkyne cycloaddition (CuAAC) reaction commonly applied in bioconjugate chemistry, in which the copper catalyst demonstrates *in vivo* toxicity.

The thiol-ene reaction may also proceed via an ionic mechanism when the alkene is part of a Michael acceptor system. The use of dehydroalanine (Dha) in a peptide sequence provides an ideal target for Michael addition, and has been demonstrated as a handle for peptide modification (Zhu and van Der Donk, 2001; Bernardes et al., 2008). Furthermore, the alkene of the maleimide moiety has been utilized as a target for peptide modification in formation of the thiosuccinimide bond (Elduque et al., 2014; Forner et al., 2020). However, this review will focus exclusively on radical-mediated thiol-ene chemistry. Ionic thiol-Michael peptide modifications have been reviewed elsewhere (Hoyle and Bowman, 2010; Tang and Becker, 2014; Gunnoo and Madder, 2016).

The use of the thiol-ene “click” reaction facilitates highly efficient application with high selectivity and little side products. The use of single electron radical chemistry provides superior selectivity in the presence of unprotected amino acid functional groups, which can include both nucleophilic and electrophilic groups prone to side reactions in two-electron approaches. A potential issue with the formation of thioether linkages is their subsequent oxidation to the corresponding sulfoxide. However, in all of the works included in this review, only Hoppmann et al. reported formation of the sulfoxide, which was attributed to the proximal photoactive azobenzene moiety facilitating further redox processes (Hoppmann et al., 2011, 2012). The sulfoxide linkage may also be seen as an advantage if increased water solubility is desired.

In the development of homogenously modified peptides and proteins, chemical modification has proven an essential tool. In nature, a variety of post-translational modifications (PTMs) of peptides and proteins result in vastly increased diversity from the 20 naturally-occurring amino acids. However, biological PTM often results in non-homogeneous mixtures through non-uniform modification, posing complications for manufacturing and production (Chalker, 2013). Chemical modifications enable precise, uniform modification of peptides for study of function and development of therapeutics. One approach for such modifications involves incorporation of unnatural amino acids or modifications to facilitate bioorthogonal reactions. Incorporation of functional groups that undergo click reactions is of particular use for the aforementioned reasons. The other approach involves targeting of native amino acid functional groups. Cys and Lys residues offer the advantage of the nucleophilic thiol and amine side-chain functional groups and their use in such modifications has been reviewed (Boutureira and Bernardes, 2015; Gunnoo and Madder, 2016). Cys residues, in particular, have a relatively low frequency of occurrence in naturally occurring sequences, at 1.48% (Agouridas et al., 2017). Development of an arsenal of varied and specific methodologies for peptide modification allows chemoselective targeting of specific residues or functional groups and is therefore of utmost importance in peptide science. An additional advantage of thiol-ene chemistry over other methodologies is in the formation of the naturally occurring thioether linkage. This is in contrast to commonly utilized linkages such as the triazole formed via CuAAC or other heterocycle-based linkers (Montgomery et al., 2019; Zhang et al., 2019).

Thiol-ene chemistry has been utilized in performing some of the most common naturally occurring PTMs through chemical modification, namely cyclization, glycosylation and lipidation. In addition to cyclization, installation of non-natural staples to α-helical peptide sequences has been demonstrated through thiol-ene chemistry. Thiol-ene chemistry has been applied less extensively in other areas of peptide science such as tagging of peptides or in probes. This review will focus on the applications of thiol-ene based modifications of peptides. Applications for polypeptide modifications and in hydrogel-peptide conjugates have been reviewed elsewhere (Hoyle and Bowman, 2010; Brosnan and Schlaad, 2014; Deming, 2016).

## Cyclization and Stapling of Peptides

Peptide cyclization is a naturally occurring PTM, with cyclizations occurring between *N*- and *C*-termini, between side chains or between the side chain and either the *N*- or *C*-terminus of the peptide chain. Cyclization is an approach often undertaken in improving therapeutic properties of peptide drugs. This is often particularly aimed at stabilizing the active conformation of the peptide, as cyclization imparts conformational constraint which reduces the entropic cost of binding, resulting in stronger, more selective target interactions (Zorzi et al., 2017; Vinogradov et al., 2019). A further advantage often observed is improved proteolytic stability, allowing improved *in vivo* half-life of therapeutics (Byk et al., 1996; Hess et al., 2008).

Cyclic peptides have been in therapeutic use since the mid Twentieth century, with the use of Gramicidin S for treatment of infected wounds in the Second World War (Gause and Brazhnikova, 1944). Octreotide presents an example with extensive modern use in oncology, with sales of USD 1.585 billion in 2019 under the brand name Sandostatin (Jón Tryggvi Njardarson, 2000; McGrath et al., 2010).

Thiol-ene chemistry has been utilized in peptide cyclization and stapling in both one- and two-component systems. Existing methods for one-component macrocyclizations involve activated alkenes or long aliphatic alkene side chains, whilst two-component stapling methodologies use dienes of varying length with native Cys residues. Examples involving thiol-yne reactions are sparsely studied and therefore beyond the scope of this review.

### One-Component Systems

One-component systems for peptide cyclization make use of at least one unnatural or modified amino acid residue, along with a complimentary functional group located on the side-chain of another amino acid that may or may not be proteinogenic (e.g., Cys, Lys). This facilitates control of the macrocyclization reaction through the introduction of the necessary amino acids or modifications in linear peptide synthesis. The direct reaction of these side chain functional groups results in macrocycle formation, with no introduction of additional linkers. The main potential issue with one-component systems is in the formation of dimers, where the intermolecular reaction occurs. This has been overcome primarily through use of dilute conditions and on-resin cyclizations which benefit from a *pseudo*-dilution effect.

**Figure 2:**
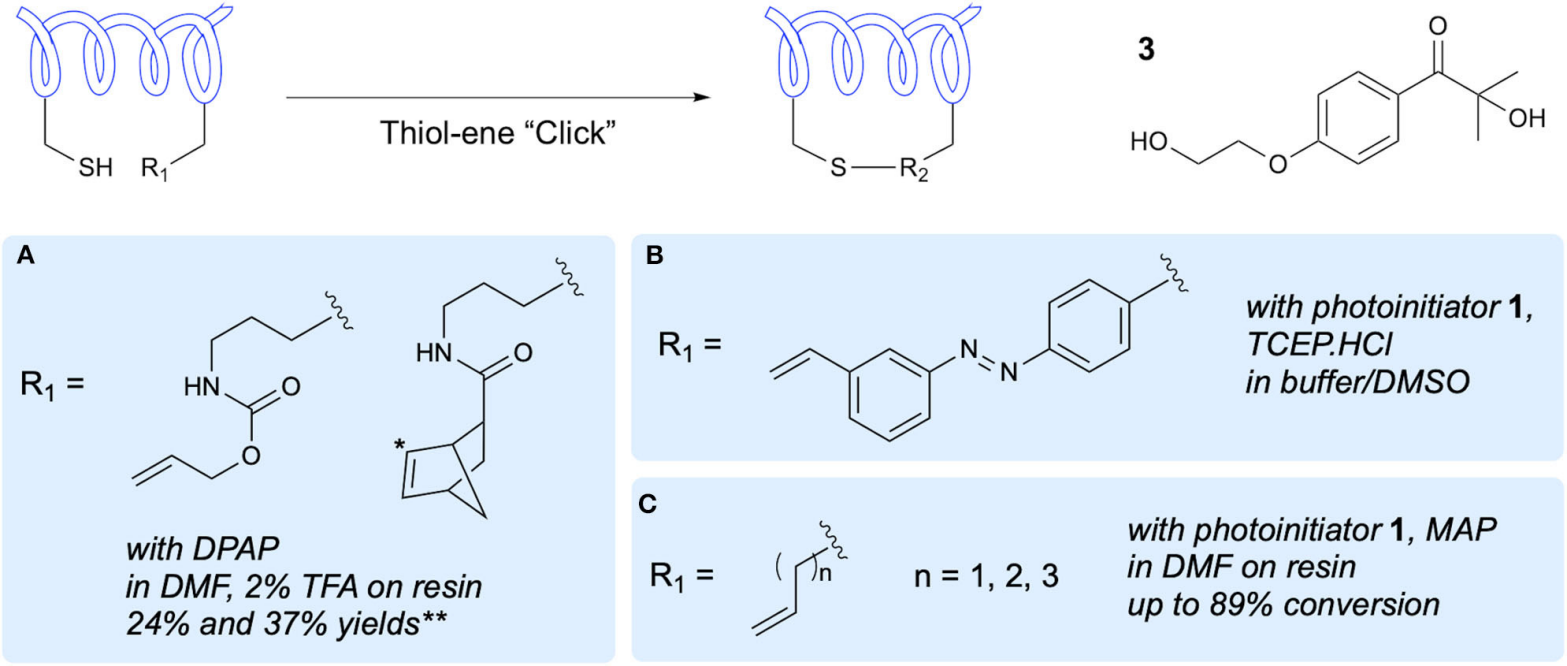
One-component peptide stapling/cyclization. Alkene-containing side-chains and side-chain modifications that have been used to demonstrate peptide stapling and cyclization and the conditions reported. *Prochiral C center at which thiyl attack takes place resulting in diastereoisomers. **Yield based on initial resin loading. **(A)** Anseth's activated alkenes. **(B)** Photoswitchable linker. **(C)** Simple alkene side chains.

In 2010, Aimetti et al. published the first reported methodology for on-resin macrocyclization of peptides via thiol-ene reaction (Aimetti et al., 2010). To incorporate an alkene group into the linear peptide sequence, a Lys side chain amino group was modified with either an allyloxycarbonyl (alloc) group or a norbornene group (Figure 2A). These functional handles were reacted with the thiol groups of Cys residues through photoinitiated thiol-ene reaction using the type I photoinitiator DPAP in DMF. This macrocyclization approach gave yields of 24 and 37% for the alloc and norbornene precursors, respectively. It was noted that the norbornene-containing peptide underwent cyclization in as little as 20 min due to the heightened reactivity of the alkene resulting from the strained bicyclic structure. NMR spectra of the norbornane-containing cyclic peptide showed diastereotopic protons at the cysteine β-position which were attributed to formation of *endo* and *exo* conformations at the norbornane. The authors investigated the thermal initiation process but found that even with extended reaction times, lower yields were achieved. Additionally, the solution-phase reaction was only briefly investigated due to the requirement for additional purification steps and work up, which gave reduced yields. The Arg-Gly-Asp (RGD) cyclic peptides produced using this methodology were evaluated for bioactivity, demonstrating improved activity upon cyclization.

A further demonstration of the use of thiol-ene reaction between a Cys thiol and the alkene of an alloc *N*-protecting group in peptide macrocyclization was carried out by Levalley et al. in tandem with strain-promoted azide-alkyne cycloaddition for additional conjugation (Levalley et al., 2018). As in the previous work, this study focused on an RGD peptide containing the Cys and alloc groups for cyclization as well as an azide for subsequent functionalization. The authors reported low yields when the cyclization was attempted on resin and so a solution-phase approach was adopted using the lithium phenyl-2,4,6-trimethylbenzoylphosphinate (LAP) photoinitiator in water. This approach gave cyclized peptide in yields of approximately 70%, and importantly retained the azide functionality. This azide group was then used for hydrogel and fluorophore conjugation.

Application of the thiol-ene reaction for installation of photoswitchable linkages in α-helical peptides was demonstrated by Hoppmann et al. using a photoswitchable click amino acid (PSCaa) containing both an azobenzene unit and an alkene handle (Hoppmann et al., 2011, 2012). Incorporation of this PSCaa into the linear sequence along with a Cys residue preceded cyclization in solution through irradiation in the presence of the photoinitiator 2-hydroxy-1-[4-(2- hydroxyethoxy)phenyl]-2-methyl-1-propanone **3**, along with *trans*-to-*cis* isomerization of the azo group (Figure 2B). The authors further investigated the cyclization reaction in presence of Cys-containing glutathione (GSH), demonstrating a preference for the intramolecular thiol-ene reaction over the intermolecular reaction of the PSCaa alkene with GSH. The authors also made the unique observation of formation of the sulfoxide linkage, previously unreported in thiol-ene reactions of peptides. This is assumed to have occurred due to further light-induced redox with the proximal azobenzene group, and provides increased solubility in aqueous media.

The work of Zhao et al. demonstrated on-resin peptide macrocyclization via thiol-ene reaction between Cys and terminal alkene bearing side chains of four to six carbons in length (Zhao et al., 2016). After optimization of the reaction conditions using a model pentapeptide bearing only Ala and the desired Cys and unnatural amino acid residues (Figure 2C), sequence compatibility was investigated through variations of the pentapeptide model to include Arg, Glu, Gly, Lys, Pro, Trp, and Tyr in varying combinations with four and five carbon alkene side chain bearing unnatural amino acids. In order to demonstrate potential for biological applications, along with compatibility with longer peptides, an *i,i*+*4* stapled analog of the 11-mer linear peptide ligand that binds the co-activator binding site of estrogen receptor was synthesized. In a fluorescence polarization assay, the cyclic peptide analog showed higher binding affinity than the linear peptide, whilst both synthetic peptides have higher affinities than the native ligand.

The one-component peptide cyclization/stapling methodologies discussed demonstrate the potential for use of thiol-ene chemistry for synthesis of cyclic peptides either on-resin or in solution as well as its utility in installation of either simple aliphatic linkers or more specialized linkers with additional functionality. A notable advantage is the need for one unnatural amino acid at most, as Cys serves as a reactive partner. However, this raises the question of selectivity where a peptide chain has more than one Cys residue. Little investigation has been performed on this potential caveat through either selectivity studies or protecting group chemistry. Nevertheless, one-component thiol-ene chemistry provides rapid access to cyclic peptides via “click” chemistry and shows potential for further study in selectivities and linker variation, aided in part by the orthogonality of the thiol-ene reaction.

### Two-Component Systems

Two-component peptide stapling involves the reaction of the peptide substrate with an external linker or staple bearing two functional groups, each with complementary reaction partners on the peptide chain. Often, the amino acids in the stapling positions must be replaced with one bearing a side chain with a suitable reaction partner for the staple. Cys is a common choice as a stapling site due the potential for reaction of the thiol group with a suitable electrophile. Additionally, as is the subject of this section, the formation of radicals at the Cys sulfhydryl can facilitate reaction with external staples. The primary challenge with two-component systems is the formation of side products, whereby the peptide chain reacts with two different linkers molecules to form a *bis*-adduct. This has been overcome through various methods that favor the intramolecular reaction over the intermolecular variant, such as selection of reactive amino acid positioning in relation to the linker or preorganization of the peptide chain.

**Figure 3:**
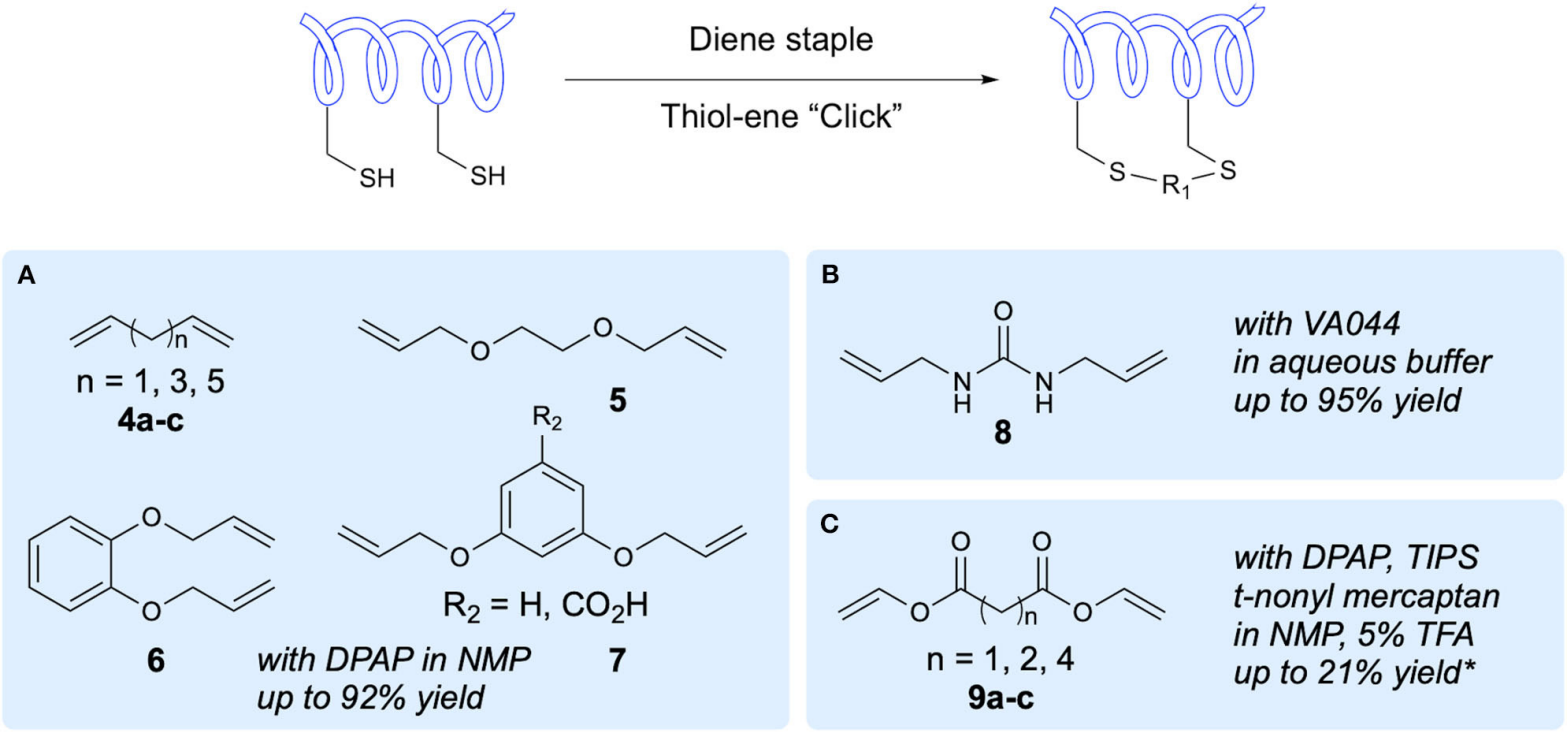
Two-component peptide stapling. A range of staples that have been applied in two-component peptide stapling via thiol-ene reaction and the conditions reported. *Yield based on initial resin loading. **(A)** Staples used in Chou's initial study. **(B)** Chou's water-compatible stapling. **(C)** Brimble's divinyl diester staples.

**Figure 4:**
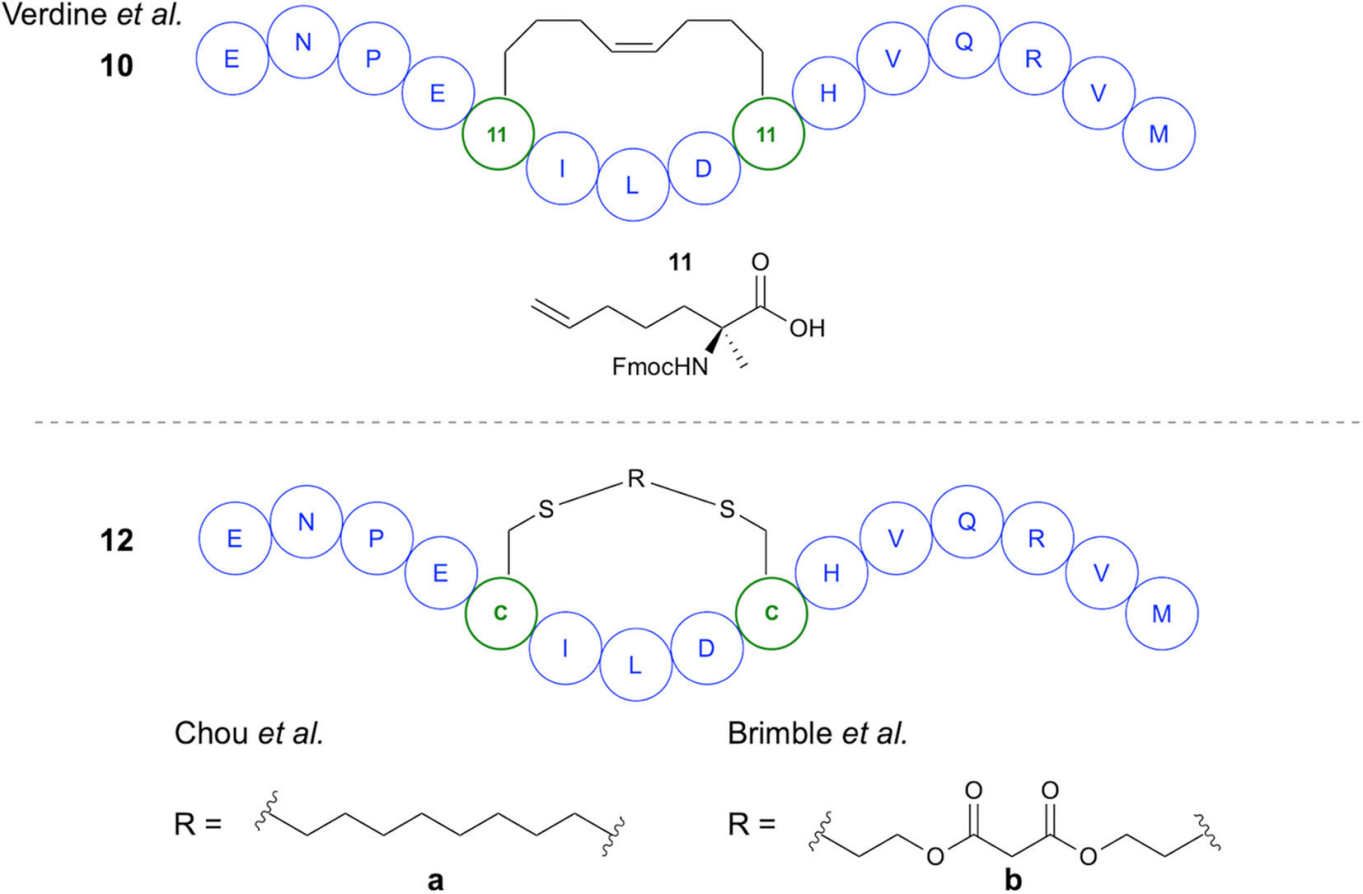
Stapled Axin mimetics. Comparison of structures of axin mimetics developed using RCM and using thiol-ene chemistry.

The first demonstration of two-component peptide stapling using thiol-ene chemistry was reported by Chou in their study of the use of thiol-ene chemistry for stapling of peptides at two Cys residues using diene staples (Wang and Chou, 2015). Importantly, this study showed the use of thiol-ene chemistry in macrocyclization of native, unmodified peptide sequences. Initial investigation of reaction conditions focused on the reaction between 1,7-octadiene and *N*-acetyl-Cys methyl ester in DMF, with DPAP proving to be the most suitable radical initiator (Figure 3A). It was subsequently found that for peptide substrates use of *N*-methyl-2-pyrrolidone (NMP) as a solvent offered improved yields. The stapling reactions for peptide examples using the DPAP photoinitiator in NMP gave yields of up to 92% for a variety of diene lengths and heteroatom substitutions for *i,i*+*4* and *i,i*+*7* Cys arrangements. To investigate the application of this stapling methodology in biologically active peptides, an *i,i*+*4*-stapled Axin mimetic **10** developed by the Verdine group using ring-closing metathesis (RCM) (Grossmann et al., 2012) was synthesized, along with the dithioether-stapled analog (Figure 4). Circular dichroism (CD) experiments showed comparable α-helicity in the reported peptide and newly synthesized dithioether-stapled peptide **12a**. Next, an *i,i*+*7*-stapled p53 mimetic developed by Bernal et al. using RCM (Bernal et al., 2007) was synthesized along with the equivalent dithioether-stapled peptide obtained via thiol-ene stapling. It was found that the dithioether stapled peptide obtained from reaction with 1,8-non-adiene showed similar α-helical properties to the previously reported peptide. Additionally the results of an ELISA assay for quantification of the efficacy of the newly obtained peptides in blocking p53-MDM2 interactions showed the dithioether-stapled peptides to be just as effective as the reported RCM-stapled peptides, whilst the unstapled peptide did not block the interaction. Importantly, the authors highlight the compatibility of this methodology with unprotected peptides without the requirement for unnatural amino acids. Further work by Wang et al. on this methodology has adapted this stapling reaction for aqueous conditions (Wang et al., 2017) Figure 3B. This was achieved through use of the water-soluble VA044 radical initiator in combination with water-soluble diallylurea staples. A notable achievement of this study is the use of otherwise unfunctionalised diene staples, as equivalent dihalogan synthons show insufficient reactivity.

Recently the Brimble group have applied divinyl diester staples to thiol-ene peptide stapling of Cys-containing peptides, providing access to a significantly more hydrophilic staple than traditional hydrocarbon staples (Figure 3C). Having previously developed their Cysteine Lipidation of a Peptide or Amino acid (CLipPA) methodology (see section Amino Acid and Peptide Lipidation), the authors demonstrated a comparable peptide stapling methodology. A linear Axin analog containing two Cys residues previously discussed in the work of Wang and Chou (2015) and based on the work of Grossmann et al. was synthesized (Grossmann et al., 2012) and used for optimization of reaction conditions for *i,i*+*4* stapling (Figure 4). Confirmation of successful stapling was achieved by mass shift experiments. Similar α-helical content to that of Chou's mimetic **12a** (88%) was observed in the diester stapled peptides, at 89% α-helical content. Further demonstration of *i,i*+*4* stapling was achieved in synthesis of SIGK stapled analogs. These are binders of Gβγ complex involved in the G protein coupled receptor signaling cascade. An additional Lys residue was incorporated into the sequence allowing attachment of fluorescein prior to stapling. Cell uptake was measured using BT549 breast cancer cells via confocal microscopy, showing uptake after 7 h incubation. In examination of potential for *i,i*+*7* stapling an analog of an IRS1-targeting peptide developed by Hu et al. was synthesized (Hu et al., 2017). Successful stapling resulted in an increase in α-helicity from 2 to 44%, representing the greatest improvement for the examples chosen.

As exemplified in the studies discussed, two-component stapling via thiol-ene chemistry allows successful and beneficial stapling of peptides using a variety of linkers. Whilst many other methods rely on predominantly aliphatic or unfunctionalised staples, thiol-ene methodology has been used to incorporate linkers containing ester, ether and urea functionalities. These staples impart different solubilities to traditional hydrophobic staples and therefore will impact overall peptide structure and solubility. Again, the use of Cys residues at the stapling locations is notable as no unnatural amino acids are required and Cys residues can be easily introduced in the desired position.

## Amino Acid, Peptide and Protein Glycosylation

It has been estimated that over half of all proteins are glycoproteins, while almost two thirds have potential *N*-glycosylation sites (Apweiler et al., 1999). The glycosylation of proteins *in vivo* results in a large degree of heterogeneity. As previously discussed, this heterogeneity proposes issues for use in syntheses. This has led to the requirement for chemical methods for selective glycosylation or other approaches for synthesis of glycopeptides. Thiol-ene chemistry has demonstrated utility in glycosylation of individual amino acids for use in solid phase peptide synthesis (SPPS) as well as modification of larger proteins. This has been approached using thiosugars and alkene-containing peptide chains as well as using alkene-functionalised sugars with native proteins. Additionally, the orthogonality of the thiol-ene reaction leads to potential for use in tandem functionalizations to introduce more than one different sugar modification. The general use of thiyl radicals in glycopeptide and glycoprotein synthesis was also reviewed by the Scanlan group in 2016 (McSweeney et al., 2016).

**Figure 5:**
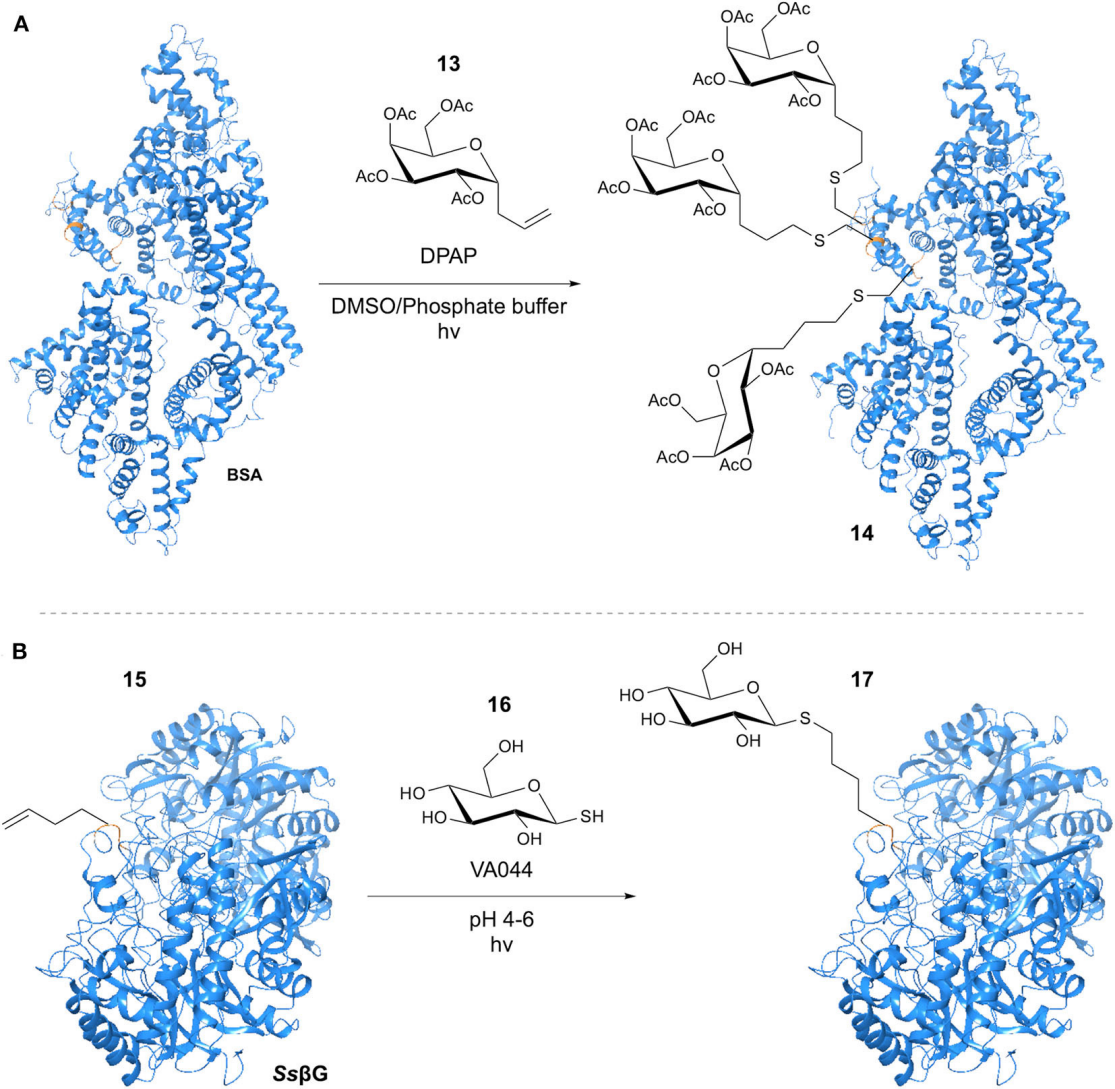
TEC-mediated protein glycosylation. **(A)** Glycosylation of the Cys residues at positions 34, 75 and 91 of BSA. **(B)** Glycosylation of Hag tag installed in β-Glycosidase.

Early work in the use of the thiol-ene reaction in glycosylation was performed by Dondoni et al. (2009), with initial investigation focusing on reaction of Fmoc-protected cysteine with a variety of monosaccharides bearing a terminal alkene-containing chain at the anomeric position. The photoinitiated reaction using DPAP in DMF with an excess of Cys provided conversion of >95%, without loss of the Cys stereochemistry. GSH was selected as a model peptide for further development of the methodology. Use of a DMF/H_2_O (1:2) solvent system facilitated glycosylation with 81% isolated yield. Application to a model nonapeptide bearing a single Cys residue was achieved via addition of a solution of the allyl α-*C*-galactoside and DPAP in DMSO to a phosphate-buffered solution of the peptide. With the goal of the study being to apply the methodology to protein glycosylation, globular bovine serum albumin (BSA) was selected as a model substrate. Modification of BSA (Bujacz, 2012) with alkene-containing glycopeptides had previously been demonstrated by Wittrock et al. (2007). Application of the conditions obtained for the nonapeptide example resulted in the addition of 3 equivalents of galactoside with cleavage of the 75↔91 disulphide bond (Figure 5A).

Also in 2009, Floyd et al. applied a “tag-modify” strategy to the thiol-ene mediated glycosylation of amino acids and proteins (Floyd et al., 2009). This involved the use of alkene-containing amino acids and a range of glycosyl thiols. Homoallylglycine (Hag) was used as a “tag” to introduce the alkene group into the protein substrate, allowing glycosylation through thiol-ene reaction with the glycosyl thiol. Initial investigation focused on single amino acid models, with the aim of optimizing the reaction for mild, aqueous conditions. To this end, the water-soluble VA044 initiator was applied at pHs ranging from 4 to 7, furnishing conversions >98% with retention of the anomeric configuration. This included a single Gal-Glc thiol disaccharide example for which >98% conversion was achieved. The authors subsequently applied their methodology to 3 model protein systems; TIM-barrell protein (*Ss* βG), (Aguilar et al., 1997) the β-helix Np276 protein and virus-like bacteriophage particle Qβ. Each protein was expressed in an *E. coli* strain, with Hag introduced in a site-specific manner through the “Met” ATG codon. Incorporation of a single Hag residue into the *Ss*βG sequence allowed reaction optimization to achieve >95% conversion at pH < 7 using VA044 (Figure 5B). Similar results were achieved for the Np276 cuboid protein, with reactions at pH 4 and 6 giving >95% conversion. For the virus-like bacteriophage particle Qβ, 180 Hag residues were incorporated. Glycosylation of all 180 alkenes was observed, with conservation of the structurally integral protein disulphide bonds and again > 95% conversion.

**Figure 6:**
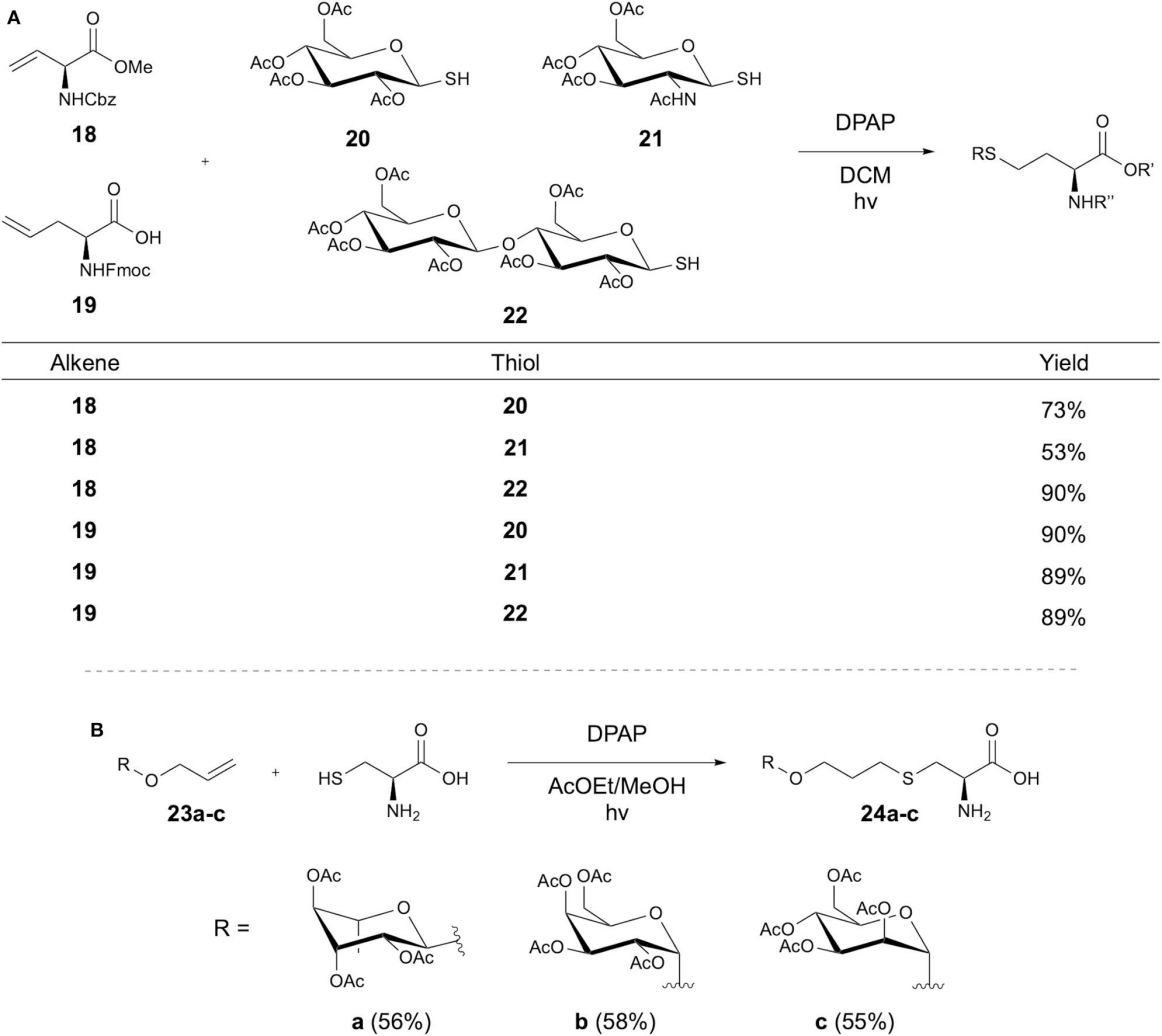
Synthesis of glycosyl amino acids via TEC. Synthesis of glycosyl amino acids achieved via **(A)** coupling of alkene-containing amino acids and glycosyl thiols or **(B)** using cysteine and an alkene-linked sugar.

Conte et al. later expanded on Conte et al. earlier studies with the double glycosylation of cysteine-containing peptides via installation of an alkyne at the Cys thiol using propargyl bromide, followed by sequential thiol-yne-thiol-ene couplings (Conte et al., 2010). In further studies, Fiore et al. reversed the functional handles of the amino acid and carbohydrate reactants, demonstrating DPAP initiated glycosylation of alkene-containing amino acids using glycosyl thiols (Figure 6A) (Fiore et al., 2011). Solution-phase peptide synthesis using a glycosylated amino acid then yielded a glycosylated tripeptide with overall 61.5% yield, demonstrating coupling at both the *N* and *C* terminus. An alternative approach to synthesis of glycosylated Cys was also presented via an initial reaction of Boc-protected Cys with 4-bromo-1-butene to give an alkene functionalized amino acid, followed by TEC with the glycosyl thiol. This approach was further applied to modification of GSH, where modification with 4-bromo-1-butene, followed by TEC afforded glycosylated GSH with 97% yield.

Conte et al. later investigated the modification of BSA first with a sugar alkyne at Cys residues via thiol-yne coupling, followed by fluorescent labeling via TEC (Conte et al., 2011). This study primarily focused on the use of thiol-yne coupling to introduce alkynes bearing sugars or other linkers.

Lázár et al. demonstrated an alternative approach through the use of enoses as the alkene component of the TEC reaction (Lázár et al., 2012). Though the majority of this work focused on non-peptidic thiols, the reaction was demonstrated using *N*-Acetyl-Cys and also the GSH tripeptide using DPAP in DMF.

The synthesis of glycosylated amino acids and their application in SPPS was also investigated by Piccirillo et al., using TEC between Fmoc-Cys and various *O*-allyl glycosides (Piccirillo et al., 2017). Elastin-derived peptides were synthesized with an *N*-terminal Cys residue, but attempts to perform TEC with the peptide gave low yields (26%), inspiring the alternative approach of SPPS using glycosyl amino acids **23a-c**. Acetyl-protected allyl carbohydrates underwent TEC with Fmoc-Cys using DPAP in an ethyl acetate/methanol solvent mix (Figure 6B). These were then applied in SPPS using racemization-reducing conditions. Cleavage from the resin followed by deprotection of the carbohydrate then followed to yield the desired glycopeptides.

The work of Rojas-Ocáriz et al. aimed to provide application of glycopeptides synthesized via thiol-ene chemistry as potential vaccines (Rojas-Ocáriz et al., 2016). The authors utilize TEC for synthesis of glycosyl amino acids for incorporation of carbohydrate antigens to MUC1 sequences via linkers of varying length to favor antigen presentation. Somewhat surprisingly, compared to previous studies, thermal initiation proved more effective, providing glycosyl amino acids with a range of linkers.

**Figure 7:**
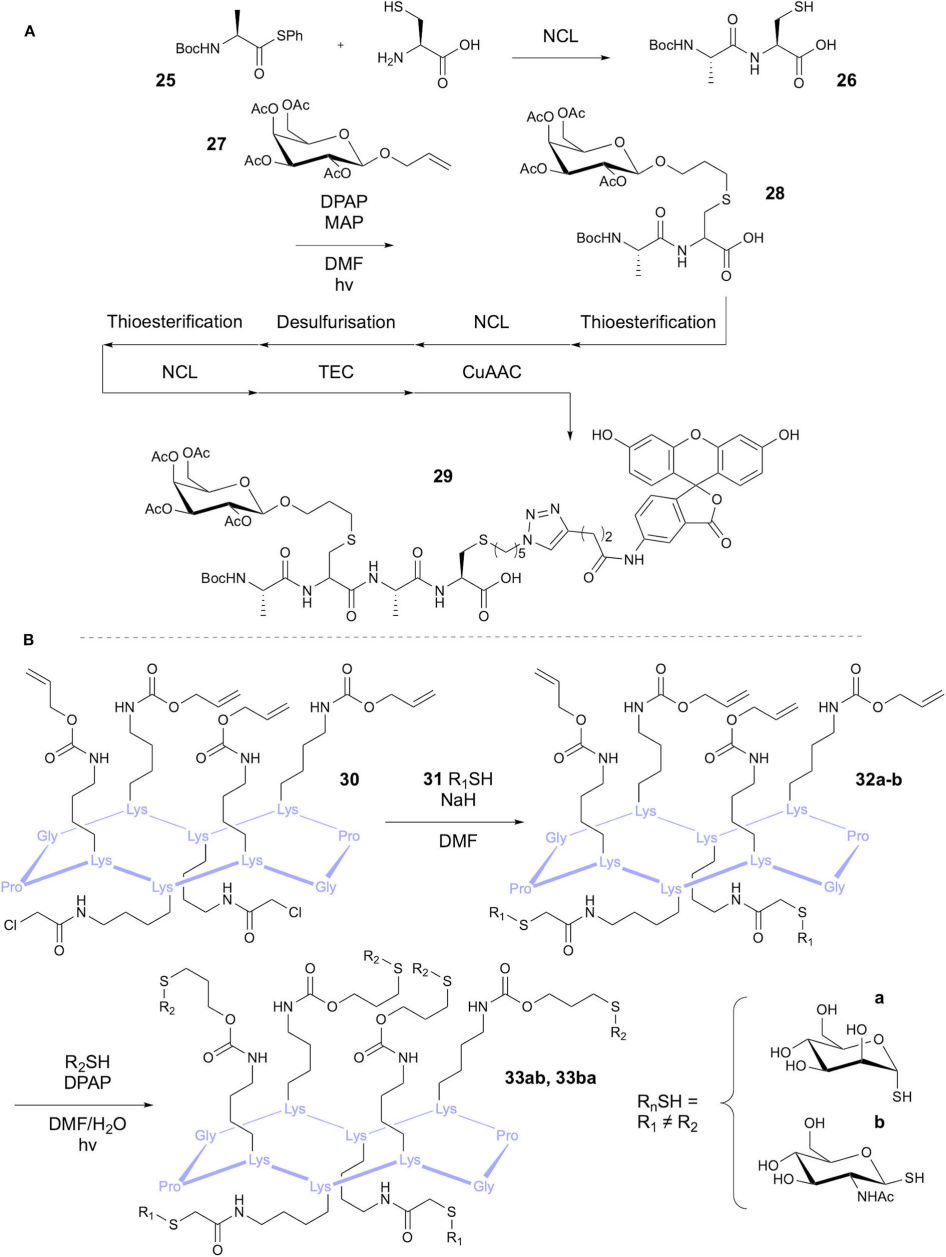
Synthesis of heterofunctionalised peptides. **(A)** Cycles of NCL and thiyl radical reactions have been used to synthesize a tripeptide with different modifications. **(B)** TEC and TCC has been used in a one-pot reaction to give heteroglycosylated peptides.

The Scanlan group has reported the modification of Cys residues re-formed after native chemical ligation (NCL) using thiol-ene chemistry (Markey et al., 2013). The aim of the study was to demonstrate three sequential NCL-Thiyl radical modification cycles to produce highly functionalized peptides (Figure 7A). The first cycle was applied to a Boc-protected alanine thioester 25 and cysteine, followed by functionalization through TEC with a glycan **27** bearing a vinyl ether group in the anomeric position. Use of the photosensitizer 4-methoxyacetophenone (MAP) and DPAP initiator afforded the glycopeptide **28** in 95% yield. Conversion of the peptide to the *C*-terminal thioester allowed a second NCL cycle with Cys, which then underwent radical-mediated desulfurization following Danishefsky's method (Wan and Danishefsky, 2007). Conversion again to the thioester allowed a third NCL, followed by thiol-ene coupling to introduce an azido group, facilitating fluorescein labeling via CuAAC. This afforded doubly functionalised tripeptide **29**, bearing a carbohydrate and fluorescein modification.

Fiore et al. applied TEC, among other approaches, to the synthesis of tetravalent glycocyclopeptides (Fiore et al., 2013). Synthesis of 10-mer cyclic peptides containing four Lys residues provided the peptidic core structure. For the thiol-ene substrates, the Lys side chains were modified with pentenyl or alloc groups, providing the alkene handle. Conversions of 82 and 85%, respectively, were achieved in coupling with the glycosyl thiol model using DPAP. This methodology was later expanded on by Fiore et al. in a one-pot procedure for synthesis of heteroglycoclusters (Fiore et al., 2014) **33a-b** via tandem thiol-chloroacetyl coupling (TCC) and TEC (Figure 7B). Orthogonality of these reactions had been previously demonstrated (Kottari et al., 2014) and therefore allowed selective functionalization of either the alloc alkene or the chloroacetyl groups using glycosyl thiols.

**Figure 8:**
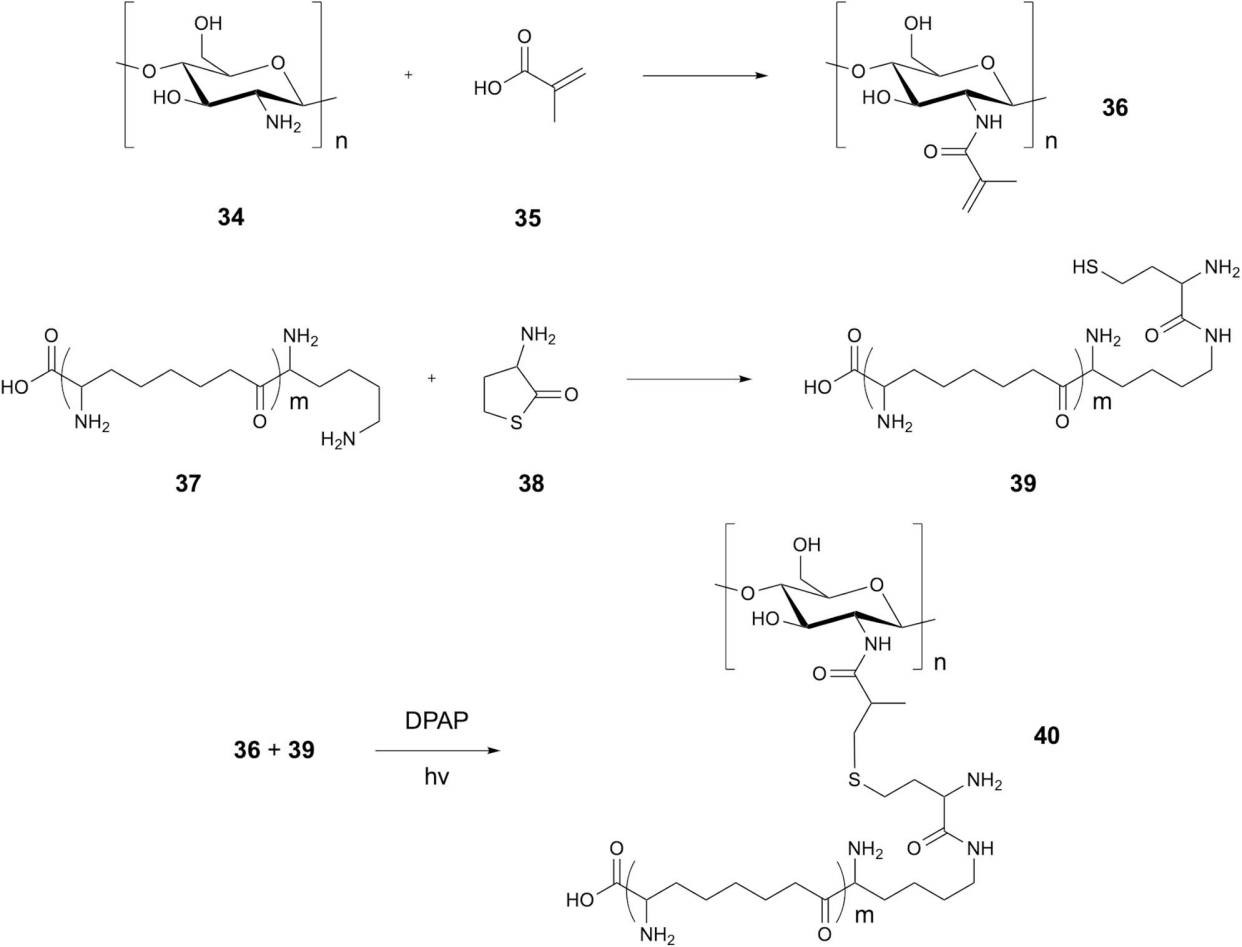
Synthesis of Chitosan peptide conjugates. Synthesis of chitosan EPL conjugates via methacrylic acid linker.

In 2017, Su et al. demonstrated the formation of a covalent attachment between chitosan and the antimicrobial peptide (AMP) α-poly-L-lysine (EPL), showing application with large polysaccharides (Su et al., 2017). Modification of the peptide *N*-terminus to include a homocysteine residue provided the thiol group, whilst modification of the chitosan amino group with methacrylic acid provided an alkene handle (Figure 8). TEC was initiated using DPAP in ddH_2_O, resulting in radical addition over possible thiol-michael coupling. Chen et al. later expanded on the utilization of thiol-ene chemistry for attachment of AMPs to polysaccharides, using thiolated polysaccharides and alkene-modified peptides (Chen et al., 2019). Thiolated dextran was coupled with aminoethyl methacrylate modified AMPs to give multiple examples of peptidopolysaccharides through TEC using DPAP in ddH_2_O.

These examples serve to highlight the broad range of approaches through which thiol-ene chemistry has been integrated in the synthesis of glycopeptides. The approach taken by Davis and co-workers in the glycosylation of 180 sites on a single protein particle allows controlled, large-scale glycosylation across proteins, utilizing a “tag-modify” strategy. In contrast, the same methodology can allow glycosylation of a single site in a protein by incorporating only one “tag.” Intermediate levels of glycosylation are also facilitated via TEC, as shown by Dondoni and co-workers using native BSA. Additionally, glycosylation has been performed using both protected and unprotected sugars. This demonstrates the range of glycosylations that have so far been achieved using thiol-ene chemistry.

## Amino Acid and Peptide Lipidation

Recombinant access to lipopeptides is severely restricted due to heterogeneity in the lipid chains, necessitating development of chemical methods for homogenous peptide modification or other synthetic methods for access to lipopeptides. Incorporation of a lipid moiety onto the peptide backbone has been shown to impart a number of therapeutically-relevant advantages such as enzymatic stability, bioavailability and membrane permeability (Hamman et al., 2005; Simerska et al., 2011; Zhang and Bulaj, 2012). Additionally, a number of lipopeptides isolated from nature show promise as potential therapeutics, including novel antimicrobial agents (Cochrane and Vederas, 2016). Approaches to chemical synthesis of lipopeptides include synthesis of lipid containing amino acids or modification of peptide chains to attach a lipid moiety, both of which have been achieved through application of thiol-ene chemistry. Modification of peptide chains via thiol-ene chemistry has been widely studied in the context of the CLipPA methodology developed by the Brimble group, involving use of fatty acid vinyl esters for attachment at Cys residues (Kowalczyk et al., 2017; Hermant et al., 2020).

**Figure 9:**
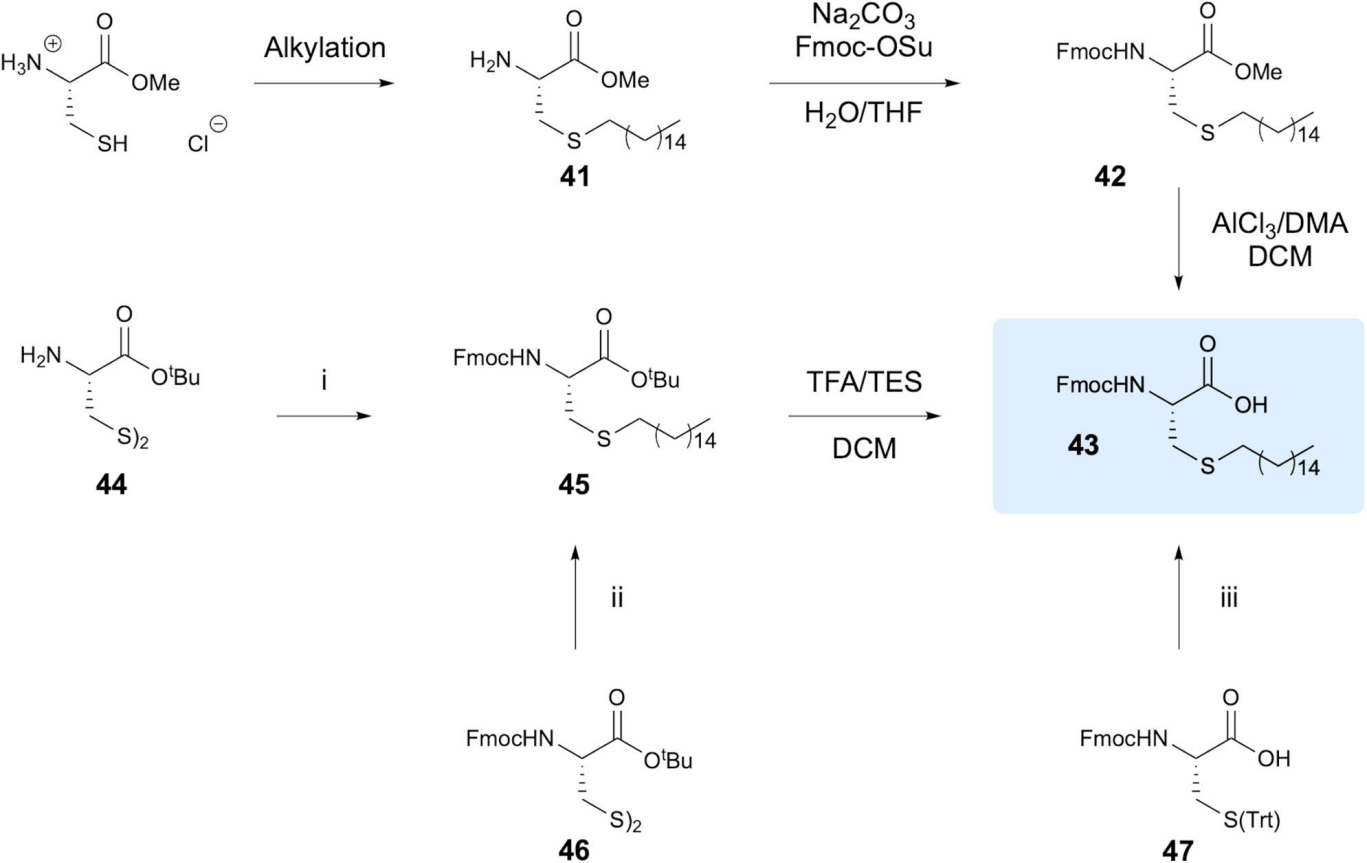
Synthesis of alkylated Cys amino acids. The various approaches to synthesis of Fmoc-Cys(hexadecane)-OH investigated are shown. (i) 1. DTT, DCM. 2. 1-Hexadecene, AIBN, DCE. 3. Na_2_CO_3_, Fmoc-OSu, H_2_O/THF. (ii) 1. DTT, DCM. 2. 1- Hexadecene, AIBN, DCE. (iii) 1. TFA/TES, DCM. 2. 1-Hexadecene, AIBN, DCE.

Triola et al. investigated the thiol-ene reaction for synthesis of lipidated Cys residues for use in preparation of lipid-modified Ras protein analogs, with particular focus on introducing non-natural hexadecylated Cys (Triola et al., 2008). The aim of the study was to provide a more efficient synthetic route to access alkylated Cys residues, as previously reported methods relying on use of basic conditions for Cys alkylation furnished yields below 30% and required extensive purification, along with significant racemization. To avoid racemization, the authors focused on the thiol-ene reaction between cysteine residues bearing varying protecting groups and 1-hexadecene, with the end goal of synthesis of Fmoc-Cys(hexadecane)-OH **43** (Figure 9). Alkylation of the Cys methyl ester provided the product in 91% yield through TEC using AIBN as the initiator, followed by Fmoc protection to give intermediate **42** in overall 74% yield. Cleavage of the methyl ester presented a problematic step, inducing racemization and resulting in an ee of only 86%. To avoid this step, the use of different protecting groups was explored. The most efficient route, giving an overall yield of 57% in four steps, employed Cystine-O^t^Bu as a starting material in which the disulphide bond was reduced using dithiothreitol (DTT), followed by TEC, Fmoc-protection and ester hydrolysis using TFA. Stereochemistry was retained to afford 99% ee in the product **36**. Various other protecting groups were also investigated, giving a range of overall yields from 42 to 55%. Notably, use of Fmoc-Cys(Trt)-OH **47** gave 55% yield in only two steps. To demonstrate wider application, **47** was reacted with a range of other alkenes including 1-octene, 2-methyl-1-hexene and *trans*-2-octene as well as a dansyl derivative and biotin marker, providing yields ranging from 35 to 91%.

Lipidation of Cys residues through TEC with vinyl esters has been extensively investigated by the Brimble group. In 2013, the Brimble group investigated the “post-translational” TEC of vinyl palmitate with Cys residues (Wright et al., 2013), noting the poor efficiency of using lipidated amino acids in SPPS. Initial investigations focused on reaction between Fmoc-Cys-OH and vinyl palmitate **48a**. Notably, thermal initiation using AIBN resulted in a complex mixture of products, whereas photoinitiation using DPAP offered an improved profile with yield of 44%. The reaction was then applied to an unprotected hexapeptide example, with thermal initiation providing no desired product, whilst photochemical conditions gave over 90% conversion via use of DPAP in NMP, with 5 equivalents of **48a**. However, significant amounts of by-products were observed due to polymerization of the vinyl palmitate. To reduce this polymerization, DTT was added to provide more efficient chain transfer via reaction with the C-centered radical resulting from thiyl radical attack on the alkene. This resulted in elimination of such polymerization by-products with conversions >90%. The reaction with a 15-mer peptide containing a terminal Cys, solubilizing polylysine tag and 9-mer epitope (CSKKKKNLVPMVATV) known to activate cytotoxic T-cells was then investigated. The lipopeptide target was obtained in >95% purity after RP-HPLC, though no yield is reported. Notably, lipopeptides prepared via this method showed comparable bioactivity to naturally occurring analogs.

**Figure 10:**
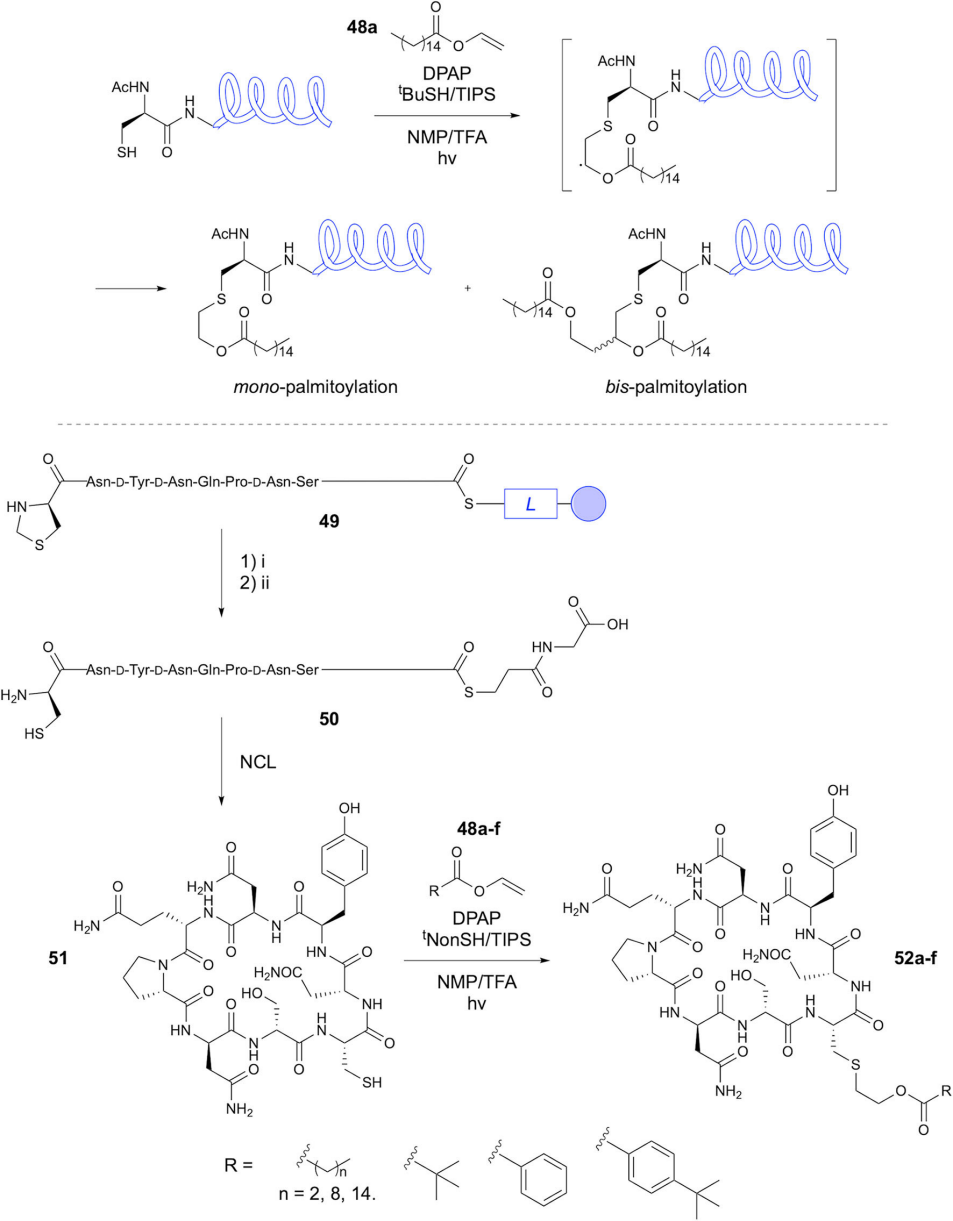
Synthesis of lipopeptides via CLipPA. CLipPA has been utilized in synthesis of linear and cyclic lipopeptides. (i) HF/*p*-cresol. (ii) methoxyamine.HCl, Na_2_HPO_4_, TCEP.HCl, pH 4.

Yang et al. later provided detailed insight into the methodology, which they termed Cysteine Lipidation on a Peptide or Amino acid (ClipPA) (Yang et al., 2016). This study aimed to vary the *N*-protecting group (Fmoc, Boc, Ac), radical initiator (DPAP, AIBN) and activation method (thermal, UV, microwave). For the Fmoc- and Boc-protected residues, photoinitiation using DPAP gave the best results at 82% isolated yield in both cases. In the case of Ac-Cys, microwave irradiation with AIBN gave the best result, with >99% yield, although other initiation conditions gave high yields of 74 to 95%. The authors then investigated the coupling of *N*^*a*^-protected *S*-palmitoylated Cys to a peptide in SPPS. To this end, a 14-mer sequence corresponding to that utilized in the previous study was constructed on-resin (SKKKKNLVPCVATV). Incorporation of Cys(^t^Bu) demonstrated tolerance for suitably protected sulfhydryl groups within the sequence. Coupling was performed using (benzotri-azol-1-yloxy)tripyrrolidinophosphonium hexafluorophosphate (PyBop) and 2,4,6-trimethylpyridine (TMP). When using the acetyl-protected amino acid a 1:1 mixture of epimers was obtained due to racemization during the formation of the activated ester. In contrast, the Fmoc protection resulted in no detectable racemization. A convergent approach for synthesis of this lipopeptide was then investigated, in which the full 15-mer sequence (AcHN-SKKKKNLVPCVATV) was constructed on resin and cleaved prior to lipidation. Optimisation of the conditions previously reported, along with addition of triisopropylsilane (TIPS) provided the desired monopalmitoylated product (Figure 10) in 88% yield, with 12% *bis*-palmitoylated by-product and overall >95% conversion. Application of the methodology to longer peptides, a 44-mer and 32-mer (AcHN-CSKKKKGARGPESRLLEFYLAMPFATPMEAELARRSLAQDA PPL-OH and H_2_N-CSKKKKVPGVLLKEFTVSGNILTIRLTAAD HR-OH), gave exclusively monopalmitoylated product in yields of 81 and 46%, respectively. The authors assign high importance to the use of the dual-reducatant ^t^BuSH-TIPS system in prevention of formation of *bis*-adducts through efficient propogation of the radical chain.

In 2018, Williams et al. reported further development of the CLipPA methodology in adaptation for on-resin lipidation (SP-CLipPA) through the synthesis of a CGRP receptor antagonist (Williams et al., 2018). This was necessitated by poor conversion and complex reaction profile when lipidation was performed in solution, as in the lipidated amino acid approach. Synthesis of the linear peptide (THRLAGLLSRSGGVVKNNFVPTNVGSKAF-RA-Resin) on resin was carried out, incorporating orthogonally protected Cys(Mmt)-OH at the *N*-terminus to allow for selective sulfhydryl deprotection. Application of previously optimized CLipPA conditions furnished the desired lipopeptide, albeit with only 20% conversion. Optimisation of reaction conditions using 70 equivalents of vinyl palmitate, one equivalent of DPAP and DMF solvent, resulted in 91% conversion, with 97:3 ratio of *mono*- to *bis*-palmitoylated product. The desired lipopeptide was obtained in 30% yield with 97% purity. This lipopeptide CGRP receptor antagonist gave equivalent antagonism to CGRP in cell studies.

Yim et al. recently applied CLipPA chemistry to the lipidation of cyclic peptides obtained via native chemical ligation (NCL) (Yim et al., 2020b). This NCL-CLipPA approach involved reaction of the Cys residue regenerated following NCL with lipid vinyl esters to afford the cyclic lipopeptide products (Figure 10). This study focused on the synthesis of an analog of iturin A, a broad spectrum antifungal cyclic lipopeptide, bearing a β-amino acid with a lipid side chain. Replacement of the α-amino acid with a lipidated Cys residue provided an analog suitable for the NCL-CLipPA synthetic approach. Boc-SPPS provided the linear peptide *C*-terminal thioester, which underwent NCL in solution upon unmasking of the d-1,3-thiazolidine-4-carboxylic acid (d-Thz) to give the *N*-terminal Cys, followed by adjustment to pH 7.5, affording the cyclic peptide **51** in 38% yield. Lipidation of the Cys was performed using vinyl palmitate **48**, TIPS/*tert*-nonylthiol (^t^NonSH), and DPAP, giving 82% conversion. This approach was subsequently applied to synthesis of six iturin A analogs **52a-f**. Cysteine modifications included short (propyl), medium (nonyl) and long (palmityl) lipid chains, a branched (^t^Bu) chain, phenyl group and *para*-^t^Bu substituted phenyl group attached via the ester linkage. Conversions achieved for the CLipPA step were high, ranging from 81 to 85%. Unfortunately, the analogs prepared did not show biological activity. The authors hypothesize that this is due to the smaller macrocycle compared to iturin A. However, this is an important demonstration of the use of natural amino acids and modification of the peptide chain to negate the challenging synthesis of chiral lipid-containing amino acids for synthesis of lipopeptides.

Yang et al. further investigated the application of alternative thiol radical chain propagators in their CLipPA methodology in place of ^t^BuSH (Yang et al., 2020b). This investigation was motivated by the desire to replace the volatile, odorous ^t^BuSH, of which approximately 80 equivalents are required for high conversions. The authors defined two selection criteria; that the thiol be sufficiently bulky to prevent *S*-alkylation, and that it should be less volatile than ^t^BuSH. Two alternatives were therefore investigated. Trityl thiol (TrtSH) is a solid and the radical generated is stabilized by the phenyl groups. ^t^NonSH is significantly less volatile and has sufficient steric bulk to prevent *S*-alkylation. Initial screening using TrtSH gave complicated product mixtures, whereas those using ^t^NonSH gave comparable results to ^t^BuSH. A number of examples using ^t^NonSH gave conversions mostly of >95%, with one example at 82%.

In their recent studies utilizing the CLipPA methodology, Yim et al. synthesized a number of linear Battacin analogs with varying lipid chains (Yim et al., 2020a). The previously developed system utilizing DPAP, TIPS and ^t^NonSH in NMP afforded lipopeptides in yields ranging from 1 to 32%, with >94% purity via either an *N*-terminal Cys or 3-mercaptopropionate (MPA) handle. Antimicrobial properties of the synthesized peptides were evaluated via minimum inhibitory concentration (MIC) values against *E. coli, P. aeruginosa*, and *A. calcoaceticus* Gram-negative bacteria, as well as Gram positive *S. Aureus*. Additionally, molecular dynamics (MD) simulations were used to study the mechanism of action. The MPA-linked lipopeptides were found to possess MICs equal to or lower than their Cys-linked counterparts bearing the same lipid. Lowest MICs were observed for the peptides baring long alkyl chains (9 carbons) or substituted aromatic moieties. This study is an important demonstration of use of the CLipPA methodology to afford a library of biologically active synthetic peptides. The group also published a study on synthesis of Paenipeptin C' analogs bearing a range of lipid moieties via CLipPA using the MPA handle (Tong et al., 2020). A number of analogs obtained showed comparable or improved activity to Paenipeptin C' against *E. coli* and *S. aureus*, again demonstrating the utility of the CLipPA methodology in preparation of a library of biologically active synthetic lipopeptides.

The CLipPA methodology was also applied to the synthesis of Connexin 43 channel inhibitory Peptide5 analogs (Yang et al., 2020a). Peptide5 moderates hemichannels and gap junctions, with implication in the progression of neurological disease. It was hypothesized that addition of a lipid moiety would help in anchoring of the peptide to the cell membrane, thus aiding function. Six peptide thiol analogs were synthesized through use of a Cys handle in place of a single native amino acid residue. One further peptide thiol was synthesized via capping of the *N*-terminus using 3-mercaptopropionic acid to provide the thiol handle. The native Cys residue in the 3-position was protected using a temporary acetamidomethyl (Acm) group, which was removed after *S*-lipidation. Previously optimized conditions for the CLipPA methodology were applied to synthesis of a total of 42 lipopeptide analogs. Biological evaluation of these lipopeptides showed no improvement in half-life compared to the natural Peptide5, but did exhibit improved functional efficacy where short lipid chains were used. This study importantly demonstrated further that the CLipPA methodology can be used in preparation of biologically active lipopeptides.

The most recent demonstration of the use of the CLipPA methodology from the Brimble group was in the synthesis of truncated *S*-lipidated teixobactin analogs (Yim et al., 2020c). Capping of the *N*-terminal of truncated teixobactin with 3-mercaptopropionic acid provided the thiol handle for CLipPA. Lipids of varying length were attached via this methodology, but unfortunately no activity was observed against *S. aureus*. It was hypothesized that the lack of activity was due to the increased hydrophilicity provided by the sulfur-containing bridging unit when compared to the control analog used, lipobactin, in which the lipid is attached directly to the *N*-terminus.

The studies discussed herein illustrate the two approaches to lipopeptide synthesis via thiol-ene chemistry, namely through incorporation of lipidated amino acids or via modification of peptide sequences. The CLipPA methodology has been extensively studied and developed, providing access to examples of biologically active linear peptides, as well as methodology for synthesis of cyclic lipopeptides. The methodology has shown compatibility with a range of sequences, producing high conversions, whilst on-resin modifications allow for greater ease of purification.

## Other Functionalizations and Applications

Thiol-ene chemistry has been used for a number of functionalizations that do not fall into the previous categories. Whilst many biological applications particularly necessitate cyclization, glycosylation and lipidation of peptides and proteins, other synthetic modifications provide useful applications for chemical biology. Modifications including attachment of small alkenes can be used as handles for further conjugation or study of peptide binding. Innovative approaches to biological studies have also incorporated radical thiol-ene chemistry for the study of protein modifications and enzyme activity.

**Figure 11:**
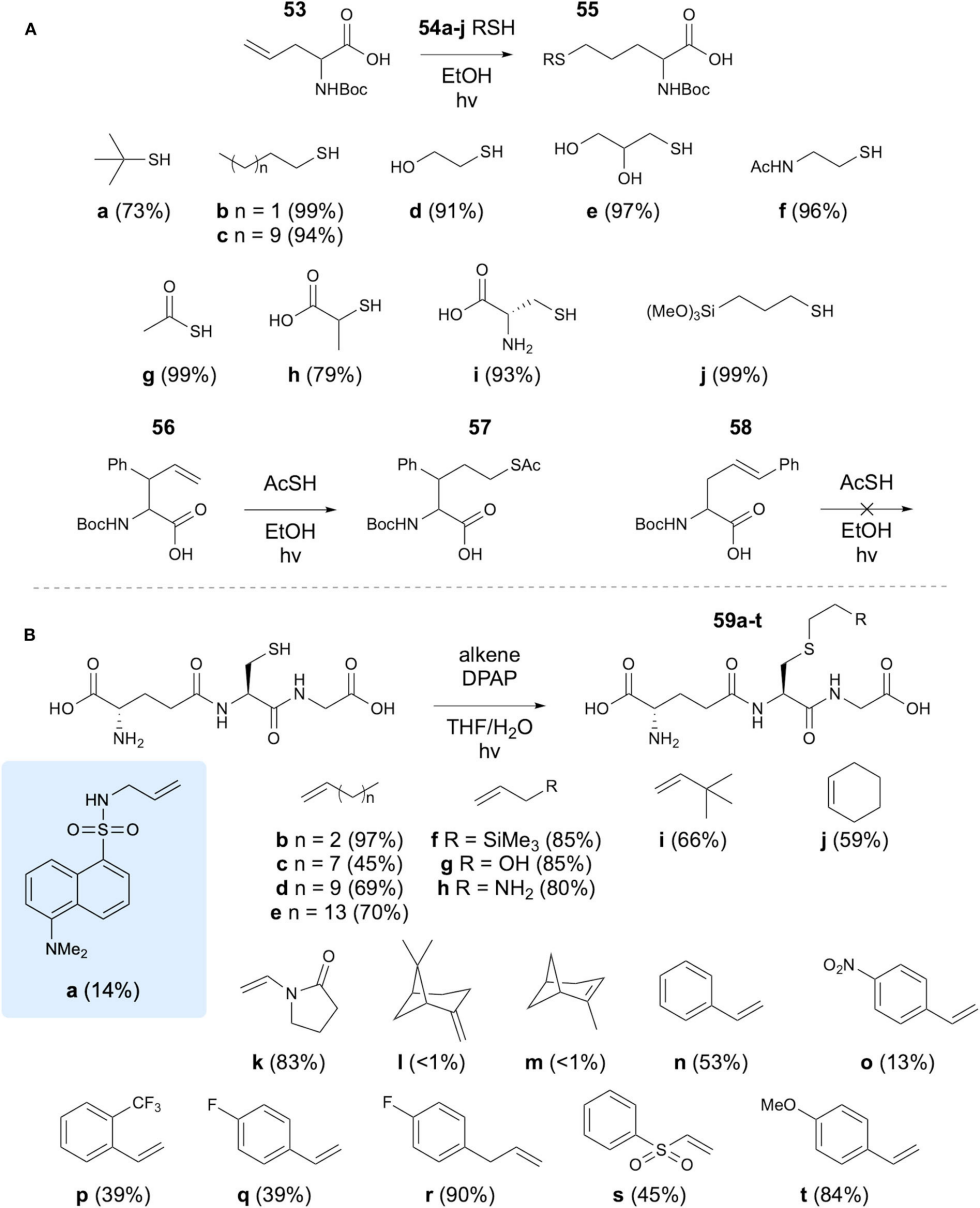
Modification of both Cys residues and unsaturated amino acid residues with small alkenes has been demonstrated via thiol-ene chemistry. **(A)** Modification of allylglycine using thiols. **(B)** Modification of Cys using small alkenes.

Karmann and Kazmier have investigated the use of TEC for the synthesis of modified amino acids, using allylglycine and a variety of thiols (Karmann and Kazmaier, 2013). Initial studies utilized Boc-protected allylglycine, which was reacted with a range of thiols using EtOH as a solvent under UV irradiation. High yields, mostly over 90%, were achieved and thiols bearing simple alkanes, alcohols, carboxylic acids, amines and a trimethoxy silicate group were coupled, along with thioacetic acid and Boc-Cys. The reaction was then applied to two further substituted alkene containing amino acids **56** and **58** (Figure 11A). The terminal alkene still provided comparable results to allylglycine, but the linear side chain amino acid did not react. The authors suggest that this is a steric effect. Investigation of the effect on the enantiomeric purity was carried out, showing reduction from 86 to 80% ee in the TEC reaction. This was further investigated in dipeptides, where no epimerization was detected. Furthermore, an allylglcine containing tetrapeptide and a tetrapeptide containing a vinyl ether side chain amino acid were successfully reacted with a selection of thiols.

Healy et al. have applied TEC to the synthesis of glutathione conjugates for study of binding to bacterial glutathione-binding protein Kef (Healy et al., 2016). Initial motivation for this study involved the synthesis of a dansyl labeled GSH analog **59a**. To this end, dansyl chloride was reacted with allylamine to introduce an alkene group, which was subsequently reacted with the Cys thiol of GSH using DPAP initiator in THF/H_2_O solvent mixture. It was found that TCEP.HCl was also required as a reducing agent to prevent formation of the disulfide, though for simpler alkenes this was excluded. A variety of alkenes were coupled with GSH with varying yields (Figure 11B). Analogs synthesized using this approach were subsequently investigated in binding studies.

Recently, the Scanlan group applied radical TEC coupling to dehydroalanine (Dha) for synthesis of *N*-terminal cysteinyl thioesters suitable for *S, N*-acyl transfer (Petracca et al., 2019). Radical TEC between *N*-protected Dha and either thioacetic acid or a glycine thioacid was effected using DPAP and MAP in aqueous buffer at pH 6. An azido Dha derivative was also utilized in reaction with thioacetic acid with full conversion, having previously failed to react under ionic conditions. The doubly Boc-protected Dha was found to be the most efficient Dha derivative in the radical process, whilst derivatives protected with a single Boc group or with a Boc and Ac group gave no conversion.

Valkevich et al. have reported the use of thiol-ene chemistry in the synthesis of branched Ubiquitin (Ub) trimers for study of isopeptidase activity (Valkevich et al., 2012). Site-directed mutagenesis was used to generate Ub monomers with a Cys residue in place of a Lys, whilst the *C*-terminus of another Ub monomer was modified with allylamine, furnishing an *N*ε-Gly-L-homothiaLys isopeptide bond upon TEC. Initial studies on dimer formation found lithium acyl phosphinate (LAP) photoinitiator to be the most effective initiator. Topoisomers, linked via different Lys position mutations were produced, with varying results due to lower conversions with increasing steric bulk surrounding the position at which the radical is generated. To investigate the function of these dimers, hydrolytic cleavage of the *N*ε-Gly-l-homothiaLys isopeptide links was investigated using isopeptidases. The authors observed a nearly identical half-life for *N*ε-Gly-l-homothiaLys and naturally occurring *N*ε-Gly-l-Lys, with site selectivity maintained for different isopeptidases. TEC was then applied in the synthesis of three Ub_3_ topoisomers. Different deubiquitinases (DUBs) were applied to hydrolysis of trimers, again showing selectivity for different linkages depending on the DUB applied.

Rafie et al. have applied TEC in the synthesis of uridine diphosphate (UDP) peptide conjugates for application as β-*N*-acetylglucosamine transferase (OGT) inhibitors (Rafie et al., 2018). To this end, allyl-UDP was reacted with Cys-containing cell penetrating peptides (CPPs) through TEC. Michael addition and disulfide exchange pathways were also investigated but proved less ideal due to need for an additional electron-withdrawing group and racemization of the Cys linker, respectively. For TEC, the initiators VA044, DPAP, and MAP were investigated, with LAP giving best results. The conjugates were shown to have activity toward human OGT *in vitro*.

**Figure 12:**
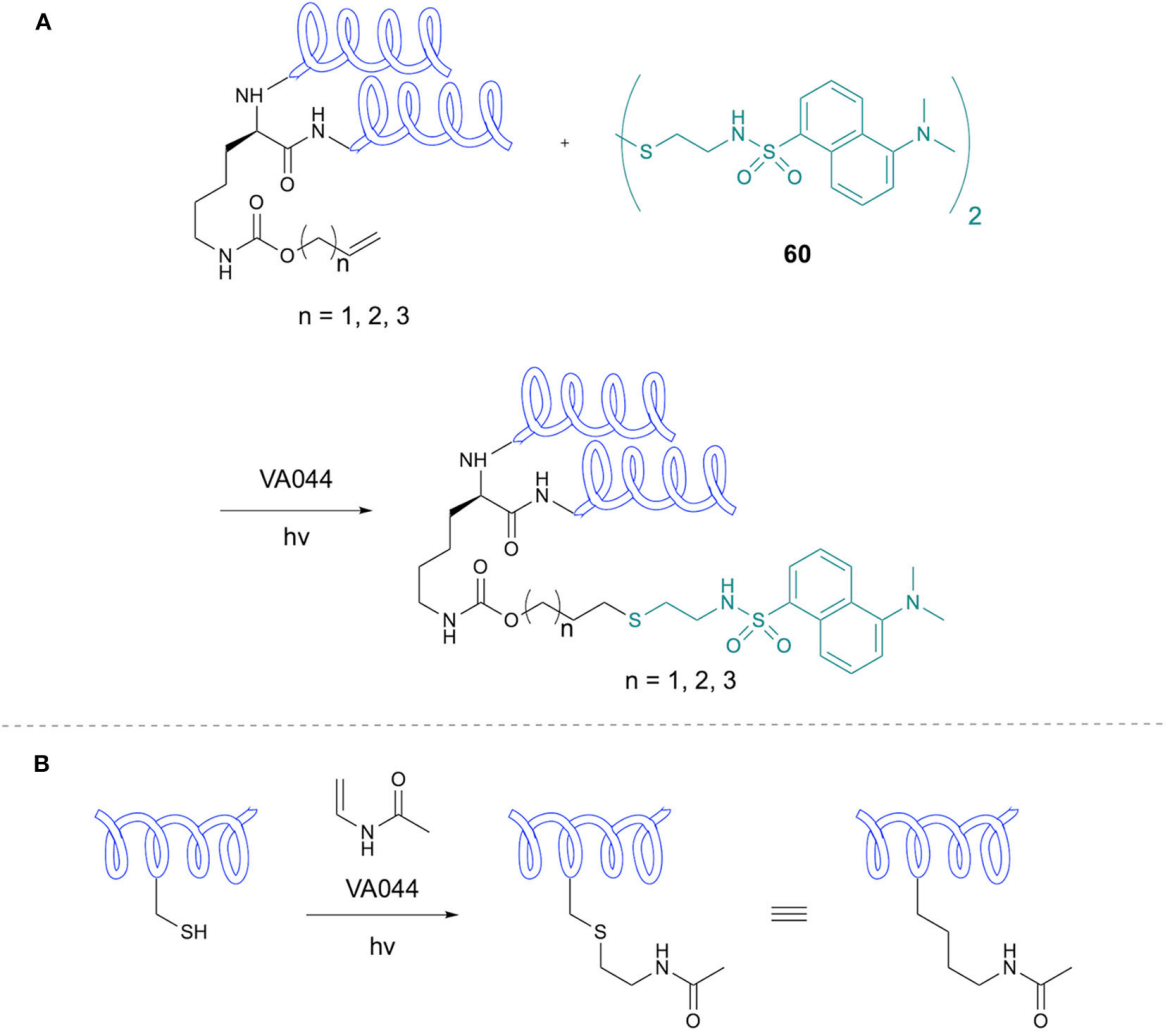
Lysine modification and Acetylated Analogs. **(A)** Synthesis of di-dansyl-tagged peptides via genetically-encoded lysine alkene modification. **(B)** Synthesis of acetylated Lys analogs through thiol-ene chemistry.

Li et al. applied TEC to introduction of fluorescent dyes to proteins incorporating genetically encoded alkene handles in an example of a “tag-modify” approach (Figure 12A) (Li et al., 2012). The authors incorporated alkene-bearing lysine derivatives into protein sequences using a mutant pyrolysyl-tRNA-synthetase (PylRS). Thiol-ene modification was applied to HdeA, an *E. coli* acid-chaperone, modified with an alkene-containing residue at the 58 position. The tagged protein was reacted with bi-dansyl-cystamine using VA044 initiation in the presence of reduced GSH. Biological studies suggested a similar activity of this modified protein to its natural analog. Modification of two sites of HdeA was then studied, with successful double ligation of bi-dansyl-cystamine. To demonstrate application to larger proteins, 36 KDa asparaginase II was tagged at the 79 position and successfully modified with the dansyl thiol without effect on the protein Cys residues.

Li et al. demonstrated the application of TEC for site-specific protein acetylation, with the aim of application in study of Lys acetylation biology (Li et al., 2011). A major challenge in this field is obtaining homogeneous protein samples with acetylation of the Lys residue of interest. The authors therefore envisaged selective modification of a Cys residue via TEC to afford a *N*-acetyl-thialysine [sLys(AC)] residue to act as Lys(Ac) mimetic (Figure 12B). The methodology was applied to synthetic Cys-tagged peptides via TEC with *N*-vinyl acetamide in the presence of VA044 as initiator, along with reduced GSH to encourage radical chain transfer, giving products in near quantitative yields. Control peptides with no Cys residue did not react under the same conditions or in absence of UV or VA044. Application to a peptide containing four Cys residues gave tetra-alkylation in 95% yield. Mutant protein examples including Ub and histones were also successfully reacted with *N*-vinyl acetamide to give the desired alkylation products. The sLys(Ac) residues proved to be suitable mimetics of Lys(Ac) in biological studies.

**Figure 13:**
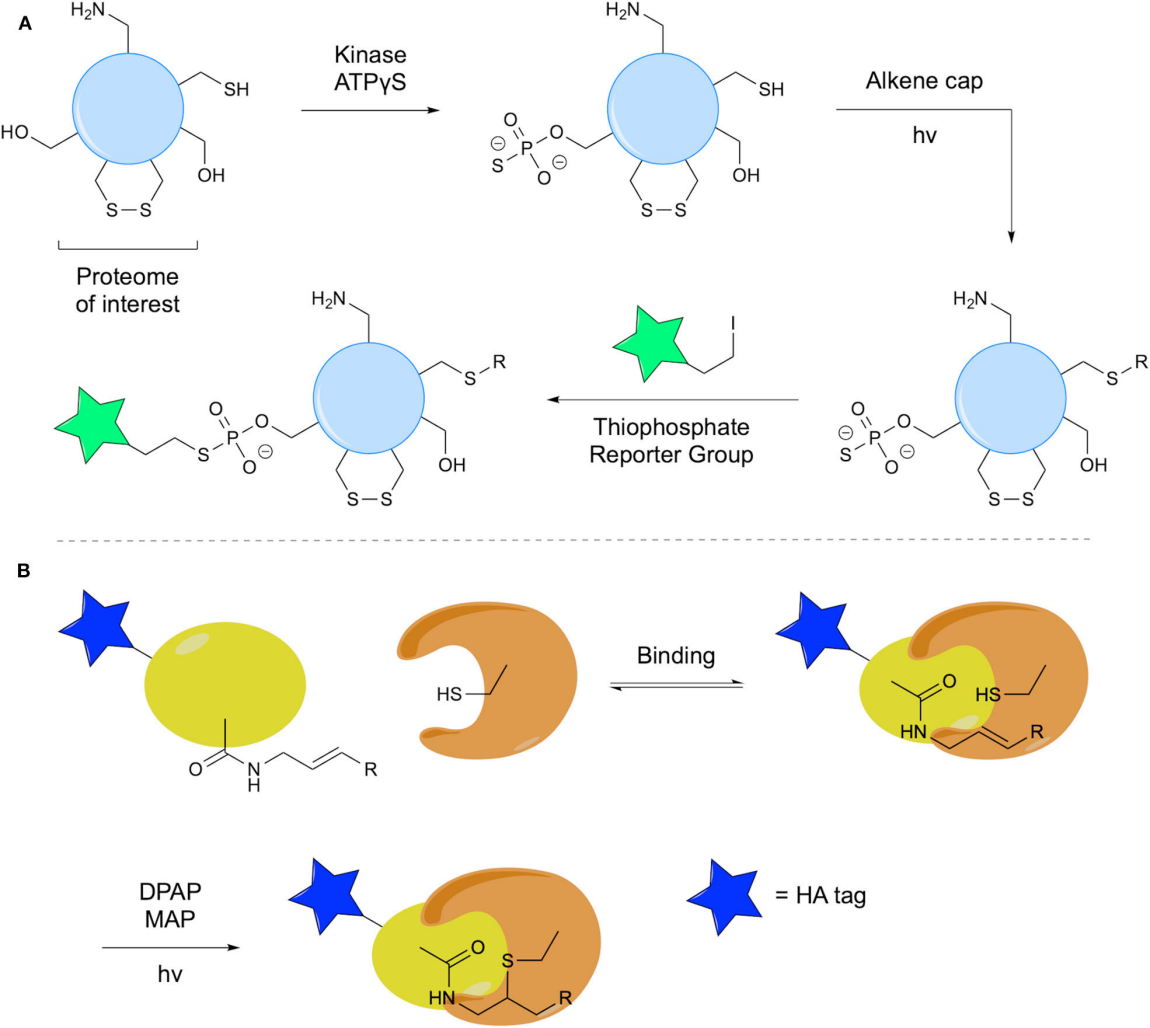
Thiol-ene in probe development. **(A)** The use of the thiol-ene reaction to cap free Cys residues in the proteome, facilitating detection of phosphorylation by a given kinase. **(B)** Use of thiol-ene chemistry in an ABP for formation of a covalent thioether linkage.

An interesting application of thiol-ene chemistry in detection of phosphorylated proteins was reported by Garber and Carlson using TEC to mask cysteine residues (Garber and Carlson, 2013). In this methodology, adenosine 5′-*O*-(3-thiophosphate) (ATPγS) facilitates transfer of a thiophosphate by an appropriate kinase. This group is subsequently employed as a nucleophile for detection of protein phosphorylation. However, to facilitate use of this nucleophilic handle, other Cys residues must be blocked. TEC was used, as it is selective for Cys thiols over thiophosphorylated residues due to differences in pK_a_, facilitating selective radical formation. In the capping of the Cys thiols, 1-(2-methoxyphenyl)-3-buten-1-ol was reacted with the protein using the LAP photoinitiator. A thiophosphorylated derivative showed no reaction under the same conditions (Figure 13A). This methodology was applied to a cancer cell proteome using three different kinases, facilitating detection of thiophosphorylated species.

Taylor et al. recently applied thiol-ene chemistry in their development of activity-based probes (ABPs) to target DUBs with the additional advantage of control over initiation of the enzyme-probe reaction (Taylor et al., 2020). Radical formation occurs at an active site Cys, triggered by UV irradiation in presence of an initiator, which in turn will react with the alkene of the probe to form a covalent enzyme-probe linkage (Figure 13B). The probe used consisted of a human influenza hemagglutinin (HA) tag linked to Ub, the *C*-terminal of which was modified with a terminal alkene. This probe was tested via incubation with recombinant DUB for 1 h, followed by addition of DPAP and MAP, degassing and UV irradiation to successfully effect TEC. A denaturation experiment confirmed the necessity for specific binding interaction prior to TEC. A short irradiation time of only 1 min was required to achieve complete labeling due to the pre-organization afforded by the binding interaction. Further, a phenyl-substituted *trans*-alkene probe was synthesized to elucidate effects on reactivity and selectivity. Comparative studies with the terminal alkene probe showed reduced labeling of protein targets.

**Figure 14:**
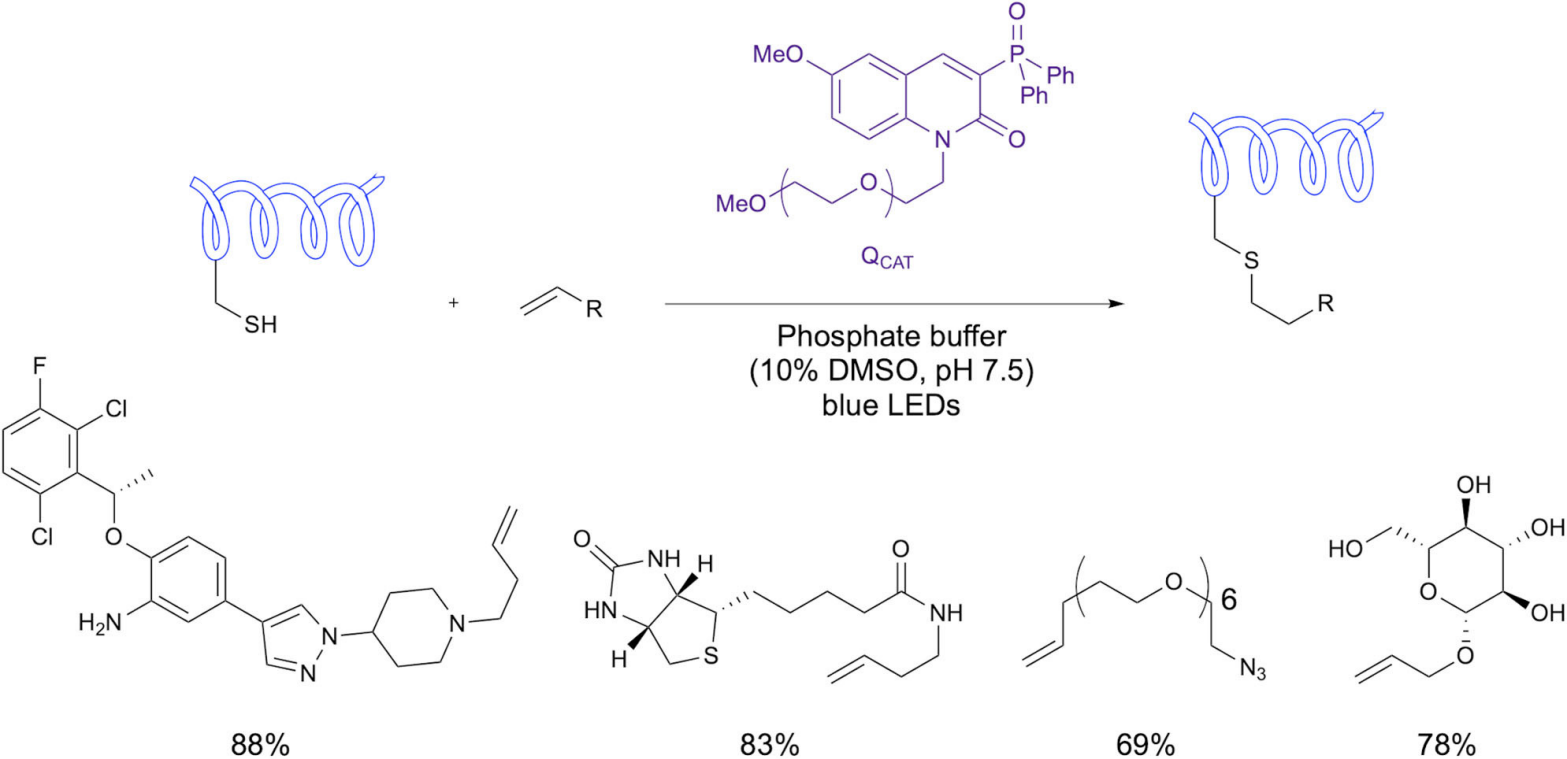
Visible light thiol-ene bioconjugation. Use of an organic photosensitizing fluorophore to facilitate bioconjugation under illumination with blue LEDS.

In a recent study from Choi et al., a water soluble fluorescent photosensitizer was utilized in visible-light-induced thiol-ene bioconjugation (Choi et al., 2020). The study demonstrated conjugation of the photosensitizer (Q_PEG_) via a PEG linker and also of biologically relevant groups (using their catalyst denoted Q_CAT_) including biotin, an azide group, a drug example and carbohydrate example (Figure 14). The reaction proceeded in phosphate buffer under irradiation with blue LEDs. Most notably, this study demonstrates the use of visible light in place of uv irradiation with both peptide and protein examples without the use of a metal catalyst.

These examples serve to highlight the broad range of applications that TEC has found in peptide and protein science, outside of the “traditional” peptide modifications of cyclization, glycosylation and lipidation. In particular, applications in biological studies show the considerable potential of innovative chemical methodologies for probing problems in biology through development of biomimietics, conjugates and probes. The “click” characteristics of the thiol-ene reaction make it an attractive tool for probe development. The highly efficient reaction, as demonstrated by very short reaction times reported by Taylor et al., allows for complete reaction with the relevant biological targets. A less efficient reaction may not provide full coverage of the groups to be investigated using the probe. Similarly, the lack of side products facilitates probe selectivity for thiol residues in targets possessing multiple varying functional groups.

## Conclusions and Outlook

Radical thiol-ene chemistry has been applied extensively to the field of peptide science. The methodology is highly compatible with sensitive biomolecules and the diverse range of functional groups found in nature. The methodology is tolerant of aqueous conditions and generally high yielding, offering unprecedented chemo- and regioselectivity in modification of peptides and proteins. Peptide stapling achieved via TEC offers considerable promise for further development of novel stapled peptides for therapeutic use, in particular with variation of the staple architecture. Glycosylation methodologies have been extensively developed, incorporating direct peptide-glycan conjugation and use of linkers. Use of the CLipPA methodology for synthesis of lipopeptides has been extensively developed. However, lipidation without use of vinyl esters is relatively poorly studied. A wide range of smaller alkenes have been conjugated to peptides through TEC for varying purposes. The use of thiol-ene chemistry in biological studies has shown particular ingenuity, demonstrating extensive potential for development of TEC-based methodologies to overcome biological problems and for biological applications. For a large number of these applications, the attraction of thiol-ene chemistry lies in its “click” characteristics. The ability to selectively react a thiol and alkene in presence of mixtures of nucleophiles and electrophiles lends itself particularly well to peptide science. The ability to incorporate unsaturated amino acids into peptide and protein sequences has been instrumental in these selective modification approaches.

Despite recent advances, thiol-ene chemistry continues to be underutilized across peptide science and in the wider context of chemical biology, certainly in comparison to the analogous Michael addition. Technical challenges associated with the photochemical nature of the process have largely been ameliorated and the methodology is now well within the means of non-specialist labs. Further innovative applications of thiol-ene in peptide science, in particular, under continuous-flow conditions, will no doubt accelerate interest in the field. The considerable advantages of this approach lend it great potential in future development of peptide modifications and applications in peptide science.

## Author Contributions

MN performed literature searches. MN and ES prepared the manuscript. All authors contributed to the article and approved the submitted version.

## Conflict of Interest

The authors declare that the research was conducted in the absence of any commercial or financial relationships that could be construed as a potential conflict of interest.
